# A case of gastropericardial fistula of a gastric tube after esophagectomy: a case report and review

**DOI:** 10.1186/1749-7922-5-20

**Published:** 2010-07-21

**Authors:** Takehito Kato, Takahiro Mori, Koki Niibori

**Affiliations:** 1 Department of Surgery, National Hospital Organization Mito Medical Center, 280 Sakuranosato, Ibaraki-machi, Ibaraki 311-3193 Japan; 2 Department of Cardiovascular Surgery, National Hospital Organization Mito Medical Center, 280 Sakuranosato, Ibaraki-machi, Ibaraki 311-3193 Japan; 3 Tohoku University Graduate School of Medicine, 1-1 Seiryo-cho, Aoba-ku, Sendai 980-8574 Japan

## Abstract

A 65-year-old man who had received an esophagectomy 10 years earlier was admitted to our hospital for right chest pain. Preoperative examinations showed pneumopericardium, a retrosternal gastric tube, and an active gastric tube ulcer. We diagnosed gastropericardial fistula of the gastric tube ulcer. Emergency surgery included lavage and drainage of the pericardial cavity and plombage of the rectus abdominis muscle flap to the posterior space of the gastric tube. Total parental nutrition and/or enteric nutrition were provided. Due to minor leakage from the ulcer, the patient could start oral intake on the postoperative 49^th ^day, and was discharged from the hospital on the postoperative 86^th ^day after physical rehabilitation. He has been free from complications for more than 33 months after surgery. Here, we review the literature and discuss the etiology and treatment of choice for this rare yet lethal complication in the follow-up after esophagectomy.

## Background

Recent advances in thoracic surgery and post-surgical management in intensive care units (ICUs) have improved the survival of esophageal cancer patients after esophagectomy; many patients often survive more than five years. However, gastric tubes that replace esophagi may erode, leading to gastric tube cancer or perforated gastric tube ulcer. Complications after gastric tube ulcer depend on the posterior-mediastinal, retrosternal or subcutaneal location of the gastric tube. Perforated ulcers of gastric tubes in the posterior-mediastinal or retrosternal spaces, if they penetrate the neighboring trachea, thoracic aorta, or pericardium, are often lethal [1-4].

We report here a rare rescued case of pericarditis due to gastropericardial fistula of the gastric tube ulcer after esophagectomy, and review 29 cases.

## Case presentation

**Figure 1:**
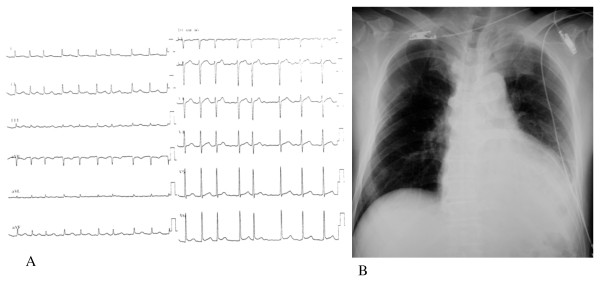
Examination on admission: electrocardiogram (A) and chest X-ray (B).

**Figure 2:**
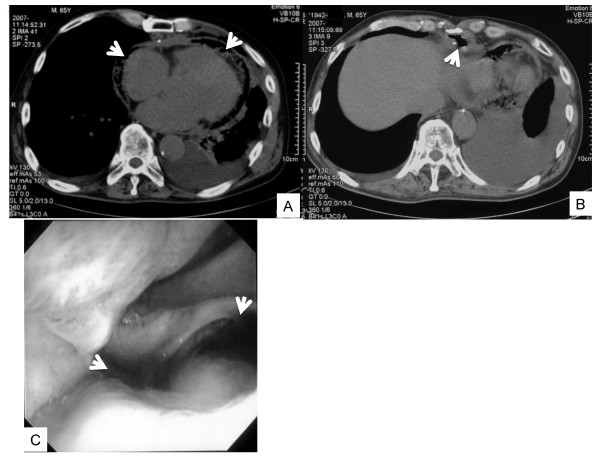
Pre-operative CT scans (A, B): arrows indicate pneumopericardium (A) or gastropericardial fistula (B); Preoperative upper GI endoscope shows the giant open ulcer within gastric tube, indicated by arrows (C).

A 65-year-old Japanese man was taken to National Hospital Organization Mito Medical Center by ambulance for severe colic right chest and back pain. He was lucid and body temperature was 36.7°C. His blood pressure was 127/97 mmHg, but atrial fibrillation (af), tachycardia, and ST-segment elevations in V5 and V6 were observed in the electrocardiogram (Figure 1A). Cardiomegaly was observed in the chest X-ray (Figure 1B). Severe inflammation was apparent, with a white blood cell (WBC) count of 9,100/μl and C-reactive protein (CRP) of 21.87 mg/dl (Table 1, left). He was hospitalized in the Department of Cardiology and conservatively treated with fluid replacement and anti-biotic chemotherapies (cefazolin). His condition worsened, with WBC and CRP increasing to 12,100/μl and 30.34 mg/dl, respectively, with liver and renal dysfunction (Table 1, right). Oxygen inhalation was required for worsening respiratory dysfunction, and he entered multi organ failure (MOF). Four days after admission, computed tomography (CT) showed pneumopericardium and a neighboring gastric tube that replaced the esophagus after esophagectomy (Figure 2A, B). The patient had a history of esophagectomy followed by reconstruction with a gastric tube via the retrosternal route for esophageal cancer 10 years previously in other hospital. One image in the whole body CT (Figure 2B) suggested the presence of a gastropericardial fistula protruding from the gastric tube and splitting the metal staples. Upper GI endoscopy confirmed an active open ulcer that penetrated the pericardium within the gastric tube at 40 cm from the incisors (Figure 2C).

**Table 1:** Laboratory data on admission and four days after admission (preoperative).

	On admission	Four days after admission (preoperative)
White blood cell (cells/μl)	9,100	12,100
Red blood cell (× 10^4^cells/μl)	304	330
Hb (g/dl)	11.1	11.8
Hct (%)	31.2	33.9
Platelet (× 10^4^/μl)	17.2	15.3
AST (IU/L)	7	2,480
ALT (IU/L)	6	903
ALP (IU/L)	200	237
LDH (IU/L)	147	2,000
Total bilirubin (mg/dl)	0.5	0.6
BUN (mg/dl)	25.5	64.9
Creatinine (mg/dl)	0.7	1.6
UA (mg/dl)	4.1	9.3
CK (IU/L)	37	44
Na (mmol/l)	138	138
K (mmol/l)	4.0	4.3
Cl (mmol/l)	102	105
CRP (mg/dl)	21.87	30.34

**Figure 3:**
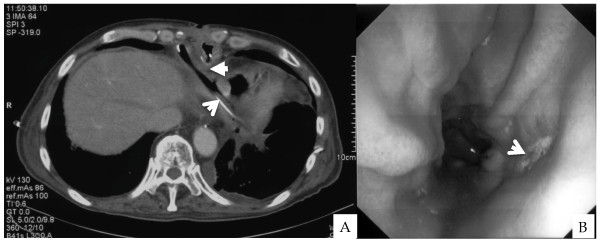
Post-operative CT shows pericardial drainage tube, indicated by an arrow, and muscular flap behind gastric tube, indicated by a triangular arrow (A); Postoperative upper GI endoscopy shows the healing ulcer, indicated by an arrow (B).

We performed emergency surgery to rescue this patient from sepsis. First, we approached to gastric tube by upper median laparotomy, given the results of CT and upper GI endoscopy. The xiphoid process and lower tip of the sternum were removed, and many adhesions were released via the right side of the minor curvature of the gastric tube to avoid injuring the right gastroepiploic artery (RGEA), which feeds the gastric tube pedicle and should be on the left side of the pedicle. We finally identified the gastropericardial fistula. A perforated ulcer of the gastric tube was detected near the bare metal staples that lined the minor curvature in the lower gastric tube, which were initially covered by seromuscular sutures as elsewhere on the gastric tube. The pericardium was opened only by releasing adhesions between the pericardium and gastric tube due to gastropericardial fistula. The pericardial abscess was saline-lavaged and a pericardial drainage tube was placed. A muscle flap was then prepared with the pedicled right rectus abdominis muscle to fill the space between gastric tube and pericardium, and wound was closed. We also drained gastric juice intermittently with a naso-gastric tube (NG tube). Post-operative CT showed the drainage tube in the pericardial space and a plombaged muscular flap between gastric tube and pericardium (Figure 3).

The pericardial abscess had already led to MOF, acute renal failure, liver dysfunction, as well as respiratory failure. Therefore, we postoperatively treated the patient in the ICU with mechanical ventilation, circulatory maintenance by catecholamines, and continuous hemodiafiltration (CHDF). For increased bilateral pleural effusion, we placed bilateral thoracic drainage tubes on the 4th post-operative day (POD). Blood oxygenation improved and he was released from mechanical ventilation on the 9th POD. On the 18th POD, gastrogram showed minor leakage from the gastric tube to the pericardium, but the drains were sufficient for pericardial drainage. He was treated with continuous pericardial drainage and nutrition support by enteric diet tube (ED tube) in the jejunum and/or by total parenteral nutrition via central venous catheter, because he sometimes experienced diarrhea with enteral tube feedings. On the 49th POD, leakage disappeared on the gastrogram, and the patient started oral intake by water drinking. On the 76th POD, gastroendoscopy showed a healing (H1) ulcer in the gastric tube (40 cm from the incisors) (Figure 3B). He was discharged from the hospital on the 86th POD, after physical rehabilitation. He has resumed daily life and is free from complications more than 33 months after surgery.

## Review of reported cases

There are only two reports of a gastropericardial fistula of a gastric tube ulcer after esophagectomy [1,5]. The other 26 cases of pericardium-penetrating gastric tube ulcers have been reported in Japan, mostly Japanese conference proceedings or case reports in Japanese. All 29 cases, including the current case, are listed in Table 2; all cases were reconstructed via a retrosternal route, except two via a posterior mediastinum, one via intra-thorax, and one unknown case. Postoperative durations vary from 2 months up to 12 years. Initial symptoms are usually chest pain or chest discomfort, with 12 patients (41%) initially presenting at cardiovascular/internal medicine or general practitioners. The current case was presented to and primarily treated by cardiologists. Conservative therapy, percutaneous pericardial drainage, or surgical drainage was adopted for 10 (37%), eight (30%), and nine patients (33%), respectively (Table 2). Thirteen patients were rescued, three in 10 by conservative therapies, two in six with trans-cutaneous drainage, including one that eventually needed additional surgical treatment, and eight in nine in surgical drainage; rescue ratios of 30%, 33%, and 89%, respectively. Prognosis in surgical drainage is much better than that in conservative therapies or in percutaneous drainage.

**Table 2:** Reported cases of gastropericardial fistula of gastric tube ulcer since 1984, quoted and partially modified from a report by Shibutani et al.

		Patient		Time between						
Case	Report year	Age	Sex	surgery and onset	Reconstruction route	Primary symptom	Initial treatment	Modality for therapy	Outcome	Reference
1	1984	46	Male	2 years 5 months	Retrosternal	Shock	Surgery	Conservative	Death	C. P.* [14]
2	1989	58	Male	3 years	Retrosternal	Chest pain, tachycardia	Internal medicine	Not described	Death	C. P.* [15]
3	1991	67	Male	3 months	Retrosternal	Precordial pain	Surgery	Conservative	Death	ref. [1]
4	1993	66	Male	9 years	Retrosternal	Chest pain	Internal medicine	Conservative	Death	C. P.* [16]
5	1993	57	Female	4 years	Intra-thoracic	Retrosternal pain	Internal medicine	Not described	Death	C. P.* [17]
6	1996	66	Male	1 year 9 months	Posterior mediastinal	Chest pain	Surgery	Conservative	Rescued	[18]
7	1997	74	Male	8 years	Retrosternal	Precordial pain	Surgery	Surgical drainage (left thoracotomy)	Rescued	[19]
8	1998	62	Male	2 months	Retrosternal	Shock	Surgery	Conservative	Death	[20]
9	1998	N/A		2 years	Retrosternal	Shock	Surgery	Surgical drainage (left thoracotomy → right thoracotomy)	Death	C. P.* [21]
10	1999	56	Male	2 years 5 months	Retrosternal	Precordial pain	Internal medicine	Surgical drainage, partial resection of gastric tube	Rescued	C. P.* [22]
11	1999	51	Male	10 months	Retrosternal	Chest pain, back pain	Surgery	Percutaneous drainage	Not described	C. P.* [23]
12	1999	68	Male	1 year 4 months	Retrosternal	Anorexia, general fatigue	Surgery	Percutaneous drainage surgical closure, partial resection of pericardium	Rescued	C. P.* [24]
13	1999	69	Male	1 year 5 months	Retrosternal	Hematemesis	Surgery	Conservative	Rescued	C. P.* [25]
14	2000	54	Male	3 years	Retrosternal	Chest pain, dyspnea	General practitioner-surgery	Percutaneous drainage	Not described	C. P.* [26]
15	2000	67	Male	5 years	Retrosternal	Precordial pain	General practitioner	Percutaneous drainage	Death	[27]
16	2000	56	Male	7 months	Retrosternal	Chest pain, shock	Surgery	Conservative	Death	C. P.* [28]
17	2003	53	Male	4 years 2 months	Retrosternal	Not described	Not described	Surgical drainage (thoracotomy), partial resection of gastric tube	Rescued	C. P.* [29]
18	2003	77	Male	4 years	Retrosternal	General fatigue	Surgery	Percutaneous drainage	Death	C. P.* [30]
19	2003	65	Male	6 months	Retrosternal	Anorexia	Surgery	Conservative	Death	[31]
20	2004	66	Male	Not described	Not described	Chest pain	Surgery	Drainage	Death	C. P.* [32]
21	2006	68	Male	2 years 6 months	Retrosternal	Chest discomfort, odynophagia	Cardiology	Drainage gastric tube resection, pericardium resection	Death	C. P.* [33]
22	2006	64	Female	5 years	Retrosternal	Chest pain	General practitioner	Surgical drainage (left thoracotomy), TachoComb^® ^sheets	Rescued	C. P.* [34]
23	2007	72	Male	4 years	Retrosternal	Chest discomfort	Cardiology	Conservative	Death	[35]
24	2008	66	Male	5 years	Retrosternal	General fatigue	Surgery	Percutaneous drainage	Rescued	[36]
25	2008	60	Male	5 years	Retrosternal	Omalgia, fever	Surgery	Surgical drainage (left thoracotomy), muscle flap plombage	Rescued	C. P.* [37]
26	2008	59	Male	12 years	Posterior mediastinal	Precordial pain	General practitionersurgery	Surgical drainage	Rescued	C. P.* [38]
27	2009	46	Female	1 year 1 months	Retrosternal	Chest pain, dyspnea	Surgery	Surgical drainage	Rescued	C. P.* [39]
28	2010	62	Male	8 years	Retrosternal	Left omalgia, melena	Internal medicine	Conservative	Rescued	[5]
29	2010	65	Male	10 years	Retrosternal	Chest pain	Cardiology	Surgical drainage, muscle flap plombage	Rescued	Current case

*C.P. = Domestic conference proceedings reported in Japanese.

## Discussion

The stomach is the organ most used for reconstructions after an esophagectomy for esophageal cancer patients; in Japan, a retrosternal route is preferred, where the gastric tube is pulled up [6]. Recent advances in surgical procedures as well as ICU care have improved the postoperative prognosis of esophageal cancer patients, but longer post-surgical periods can lead to problems with gastric tubes, such as bleeding, perforated ulcers, or gastric tube cancers. More than 13% of patients eventually have gastric tube ulcers [7], which can cause massive bleeding, perforation, or penetration through neighboring vital organs [1-4]. Gastropericardial fistula is highly lethal, with a high mortality of more than 50% (Table 2). Almost all cases were reconstructed via the retrosternal route, as the gastric tube is close to the pericardium. The blood supply for the stomach is mostly dependent on the left gastric artery (LGA), so a gastric tube without the LGA reduces blood supply by 84% at distal sites or by 40% to 52% at middle or proximal sites, where blood supply is replaced by the RGEA [8]. Blood supply also declines more in the retrosternal than the posterior mediastinal route [9]. This decreased blood flow can cause the ulcer, even in the normal healing process [10]. This case showed a thinned, weakened gastric tube wall, with simple closure of a penetrated ulcer usually insufficient. Muscle flap plombage can help treat pericardial or mediastinal abscesses, as we used here with rectus abdominis muscle for a good outcome [11-13].

## Conclusions

Esophageal cancer patients have prolonged survival after esophagectomy, but gastric tube ulcers can be life-threatening. We found that both surgical drainage and muscle flap plombage can be beneficial for treating ulcers. Gastropericardial fistula of a gastric tube ulcer should be part of the differential diagnosis in patients with an esophagectomy, especially via retrosternal route, that present with chest pain. Similarly, routine examination of the gastric tube by upper GI endoscopy could help avoid this high-mortality comorbidity.

## Consent

Written informed consent was obtained from the patient for publication of this case report and any accompanying images. A copy of the written consent is available for review by the Editor-in-Chief of this journal.

## Competing interests

The authors declare that they have no competing interests.

## Authors' contributions

TK was involved in the surgery and was a major contributor in writing the manuscript and preparing figures and tables. TM performed the emergency surgery and gave final approval of the version to be published. KN participated in the surgery team and performed pericardial lavage and drainage as a department chairman of Cardiovascular Surgery. All authors read and approved the final manuscript.
